# Mare’s Milk for Gut Microbiome Restoration and Immune Recovery After COVID-19 in Children and Pregnant Women: A Hypothesis-Generating Systematic Review

**DOI:** 10.3390/biom16081203

**Published:** 2026-08-17

**Authors:** Zhanna Rakhimbayeva, Abdujalil Mussayev, Ainash Oshibayeva, Gulnaz Nuskabayeva, Saltanat Kyrykbayeva, Lazzat Begimbekova, Saltanat Khudaibergenova, Karlygash Sadykova, Zhanar Zhagiparova, Mohamad Aljofan

**Affiliations:** 1 Faculty of Medicine, Khoja Akhmet Yassawi International Kazakh-Turkish University, Turkestan 161000, Kazakhstan; 2 Faculty of Medicine, Kyrgyz-Russian Slavic University Named After B.N. Yeltsin, Bishkek 720000, Kyrgyzstan; 3 Center for Strategic Development, Khoja Akhmet Yassawi International Kazakh-Turkish University, Turkestan 161000, Kazakhstan; 4 Department of Biomedical Sciences, School of Medicine, Nazarbayev University, Astana 010000, Kazakhstan

**Keywords:** COVID-19, gut microbiome, gut dysbiosis, mare’s milk, microbiota, immune modulation

## Abstract

Background: Mare’s milk has gained attention as a functional food due to its bioactive compounds and potential microbiome-modulating properties. This systematic review evaluated the evidence on its potential role in gut microbiome restoration and immune modulation following COVID-19, particularly in pediatric and maternal populations. Methods: PubMed/MEDLINE, Scopus, Web of Science, and Embase were systematically searched for studies investigating mare’s milk or koumiss and their effects on gut microbiota, immune responses, inflammatory markers, or gastrointestinal outcomes. Results: Eight studies were included: five examined COVID-19-associated gut microbiome alterations, and three investigated the biological effects of mare’s milk or fermented mare’s milk. COVID-19 was consistently associated with reduced microbial diversity, depletion of beneficial bacteria, and enrichment of opportunistic pathogens, with some changes persisting after recovery. Conclusions: Koumiss demonstrates biologically plausible microbiome-modulating and immunoregulatory properties that may support recovery from COVID-19-associated gut dysbiosis. However, current evidence remains indirect, and clinical studies are needed before recommendations can be made.

## 1. Introduction

The human gastrointestinal tract harbors a diverse and complex microbial ecosystem that plays a crucial role in nutrient metabolism, maintenance of intestinal barrier integrity, and regulation of innate and adaptive immune responses [1,2,3]. Disruption of this microbial balance, known as gut dysbiosis, has been associated with numerous inflammatory, metabolic, and infectious conditions [4,5]. Since the emergence of coronavirus disease 2019 (COVID-19), increasing evidence has demonstrated that severe acute respiratory syndrome coronavirus 2 (SARS-CoV-2) infection extends beyond the respiratory system and substantially influences the gastrointestinal environment and gut microbial composition [6,7].

Patients with COVID-19 commonly exhibit reduced microbial diversity, depletion of beneficial commensal bacteria such as *Faecalibacterium prausnitzii* and *Bifidobacterium* species, and enrichment of opportunistic pathogens, including *Enterococcus* and *Streptococcus* species [7,8]. Importantly, these alterations may persist for months after the acute infection and have been implicated in chronic inflammation, immune dysregulation, and the development of post-acute COVID-19 syndrome (Long COVID) [9,10,11].

Children and pregnant women represent populations in whom maintenance of a healthy gut microbiome is particularly important. During childhood, microbial colonization and maturation influence immune development, metabolic programming, and susceptibility to disease later in life [1,2,3]. Likewise, pregnancy is accompanied by dynamic and physiologically regulated alterations in maternal gut microbial composition that contribute to metabolic adaptation and immune tolerance necessary for fetal growth [4,5]. Disturbances in maternal or pediatric microbiota following COVID-19 may therefore have broader consequences, including altered immune responses, increased inflammation, and potential effects on maternal and offspring health [6,8].

Given the strong interaction between diet and microbial ecology, nutritional approaches aimed at restoring gut microbial homeostasis have received increasing scientific interest. Dietary components can alter microbial diversity and activity, particularly through modulation of short-chain fatty acid (SCFA)-producing bacteria, enhancement of intestinal barrier function, and regulation of inflammatory signaling pathways [9,10,11]. Functional foods containing prebiotic substrates, probiotics, and bioactive compounds have therefore been proposed as promising adjunctive strategies to support post-infectious microbiome recovery [10,12].

Among these functional foods, mare’s milk has gained attention due to its unique nutritional and biological properties. Mare’s milk possesses a composition that resembles human milk more closely than bovine milk, particularly because of its higher lactose concentration, lower casein content, favorable whey protein ratio, and abundance of bioactive molecules including lactoferrin, lysozyme, immunoglobulins, essential fatty acids, vitamins, and antioxidant compounds [13,14,15]. Fermented mare’s milk products such as kumis also contain lactic acid bacteria and yeasts that may exert probiotic effects by promoting beneficial microbial populations and enhancing mucosal immune responses [6,16]. Experimental and clinical studies have suggested that mare’s milk and its fermented products may improve gastrointestinal function, modulate immune activity, and influence gut microbial composition, suggesting potential therapeutic applications in conditions associated with dysbiosis [15,16,17].

The possible role of mare’s milk may be particularly relevant in children and pregnant women recovering from COVID-19, where safe nutritional interventions capable of supporting microbiome restoration are highly desirable [6,7]. However, despite growing interest in the relationship between functional foods, microbiota, and post-COVID recovery, the evidence regarding the specific effects of mare’s milk on gut microbial diversity, microbial composition, inflammatory markers, and clinical outcomes in these vulnerable populations remains limited and fragmented [17,18]. To date, no comprehensive synthesis of available evidence has evaluated the potential of mare’s milk as a microbiome-targeted intervention following COVID-19.

Therefore, the aim of this systematic review is to critically evaluate and synthesize the available evidence regarding the effects of mare’s milk and fermented mare’s milk products on gut microbiome composition, microbial diversity, immune function, inflammatory responses, and gastrointestinal health in children and pregnant women following COVID-19 infection.

We hypothesize that supplementation with mare’s milk or its fermented products may theoretically contribute to restoration of post-COVID gut microbial homeostasis by increasing beneficial commensal microorganisms, improving microbial diversity, enhancing production of anti-inflammatory microbial metabolites, and reducing persistent immune activation [9,10,11,12,17]. However, because direct clinical evidence in post-COVID-19 pediatric and maternal populations is lacking, this review aims to evaluate whether the available indirect and mechanistic evidence provides a biological rationale for investigating mare’s milk as a potential nutritional strategy in future clinical research, while identifying key knowledge gaps.

## 2. Methodology

### 2.1. Study Design

This systematic review was conducted to evaluate the available evidence regarding the effects of mare’s milk and fermented mare’s milk products on gut microbiome restoration, immune regulation, and related health outcomes in children and pregnant women following COVID-19. Given the emerging nature of this research area and the anticipated scarcity of direct clinical studies, the review also considered relevant evidence from comparable populations and experimental models to provide a comprehensive understanding of the potential mechanisms underlying the microbiome-modulating effects of mare’s milk. Thus, this review includes indirect mechanistic evidence due to absence of clinical intervention studies in target population.

The review methodology was developed in accordance with the Preferred Reporting Items for Systematic Reviews and Meta-Analyses (PRISMA) 2020 guidelines. The review protocol was prospectively registered in the International Prospective Register of Systematic Reviews (PROSPERO) (registration number: CRD420261438808).

### 2.2. Search Strategy

A comprehensive literature search was performed to identify studies investigating the relationship between mare’s milk supplementation, gut microbiome composition, immune responses, and post-infectious recovery. The following electronic databases were systematically searched from their inception until the final search date 4 May 2026: PubMed/MEDLINE, Scopus, Web of Science, and Embase. Additional records were identified through manual screening of reference lists of relevant articles and review papers.

The search strategy incorporated a combination of Medical Subject Headings (MeSH) and free-text terms related to four main concepts: mare’s milk, gut microbiota, immune function, and COVID-19. Representative search terms included “mare’s milk,” “equine milk,” “fermented mare’s milk,” “kumis,” “gut microbiome,” “gut microbiota,” “intestinal microbiota,” “microbial diversity,” “dysbiosis,” “immune response,” “inflammation,” “immunomodulation,” “COVID-19,” “SARS-CoV-2,” “children,” “pediatric,” “pregnancy,” and “maternal health.” Boolean operators (“AND” and “OR”) were applied to optimize the sensitivity and specificity of the search. The complete search strategies for each database are provided in Supplementary Table S1.

### 2.3. Eligibility Criteria

#### 2.3.1. Inclusion Criteria

Studies were eligible for inclusion if they addressed one of two complementary evidence streams. The first evidence stream comprised studies investigating COVID-19-associated gut microbiome alterations, including changes in microbial composition, diversity, immune markers, inflammatory responses, gastrointestinal health, or related clinical outcomes. These studies were included to characterize the nature and persistence of gut dysbiosis following SARS-CoV-2 infection. The second evidence stream comprised studies evaluating the microbiome-modulating or immunological effects of mare’s milk or fermented mare’s milk products (e.g., koumiss). These studies investigated changes in gut microbiome composition, microbial diversity, immune markers, inflammatory responses, gastrointestinal health, or related biological outcomes in human or experimental models.

The population of interest included children and pregnant women with a history of COVID-19; however, due to the anticipated limited availability of direct evidence, studies involving healthy pediatric or maternal populations, adult populations with COVID-19-associated gut dysbiosis, other human populations, and relevant animal models investigating the microbiome or immunological effects of mare’s milk were also considered for mechanistic and supportive evidence. Adult studies were included to provide biological and mechanistic insights into COVID-19-associated gut microbiome alterations in the absence of sufficient pediatric and maternal data. These studies were not considered direct evidence for the target populations but were used to establish biological plausibility and identify priorities for future population-specific research.

Eligible study designs included randomized controlled trials, non-randomized intervention studies, prospective and retrospective cohort studies, cross-sectional studies, and relevant experimental animal studies. Studies reporting quantitative outcomes, including changes in bacterial abundance, microbial diversity indices, short-chain fatty acid production, inflammatory cytokines, immune biomarkers, or gastrointestinal outcomes, were included. Studies providing qualitative evidence regarding microbiome modulation or immune effects of mare’s milk were also considered for narrative synthesis.

#### 2.3.2. Exclusion Criteria

Studies were excluded if they did not address either of the two predefined evidence streams: (1) COVID-19-associated gut microbiome alterations or (2) the microbiological, immunological, or biological effects of mare’s milk or its fermented products. Studies that did not report relevant outcomes related to gut microbiota, immune function, gastrointestinal health, or other predefined biological effects were excluded. Studies evaluating other dairy products without separately reporting relevant mare’s milk findings were also excluded. Review articles, systematic reviews, editorials, commentaries, conference abstracts without sufficient data, case reports with limited clinical relevance, unpublished studies, and other non-original research articles were excluded. Studies not available in full text or without adequate methodological information to evaluate their findings were also excluded.

### 2.4. Study Selection

All identified studies were imported into Microsoft Excel, and duplicate records were removed before screening. Two independent reviewers performed title and abstract screening to identify potentially relevant studies. Full-text articles of selected records were subsequently evaluated according to the predefined inclusion and exclusion criteria. Disagreements between reviewers during the screening or eligibility assessment stages were resolved through discussion and consensus. Any disagreements regarding study inclusion or exclusion were first resolved through discussion and consensus between the two reviewers. If consensus could not be reached, the third reviewer independently evaluated the study and acted as an adjudicator to make the final inclusion decision. Reasons for exclusion at the full-text screening stage were documented and are summarized in the PRISMA flow diagram.

### 2.5. Data Extraction

Data extraction was independently performed by two reviewers using a standardized and pre-designed data collection form. The extracted information included the first author, publication year, country of origin, study design, study population, sample size, participant characteristics, COVID-19 status when available, type and characteristics of mare’s milk intervention (raw, processed, or fermented products such as kumis), dosage, duration of supplementation, and follow-up period.

Additional information regarding microbiome assessment methods, including sequencing techniques, microbial diversity measurements, and reported changes in bacterial taxa, was collected. Immunological outcomes, including cytokine concentrations, inflammatory biomarkers, immune cell profiles, and other indicators of immune function, were extracted when available. Clinical outcomes related to gastrointestinal symptoms, infection recovery, nutritional status, and overall health were also recorded. All extracted data were reviewed for completeness and accuracy.

### 2.6. Quality Assessment and Risk of Bias

The methodological quality of included studies was independently assessed by two reviewers using validated tools appropriate to each study design. The Risk of Bias for non-randomized studies was performed using the Newcastle–Ottawa Scale (NOS). For experimental animal studies, the Systematic Review Centre for Laboratory Animal Experimentation (SYRCLE) risk of bias tool was used to assess methodological quality. The SYRCLE tool evaluates ten domains of potential bias, including sequence generation, baseline characteristics, allocation concealment, random housing, blinding of caregivers and investigators, random outcome assessment, blinding of outcome assessors, incomplete outcome data, selective reporting, and other sources of bias. Each domain was assessed separately and categorized as low, unclear, or high risk of bias based on the information reported in the original studies. The assessment considered factors including selection bias, allocation procedures, blinding, outcome assessment, completeness of outcome data, selective reporting, and potential confounding factors. Particular attention was given to methodological factors such as randomization and blinding, as incomplete reporting or inadequate implementation of these procedures may influence the reliability, reproducibility, and interpretation of experimental findings. Importantly, the risk-of-bias assessment was used not only to describe methodological quality but also to inform the certainty and interpretation of evidence derived from animal and laboratory-based studies. Any discrepancies between reviewers were resolved through discussion or consultation with a third reviewer.

### 2.7. Data Synthesis and Analysis

A descriptive and narrative synthesis was performed to summarize the characteristics and findings of the included studies. Studies were categorized according to population (children, pregnant women, adults, and experimental models), COVID-19 status, type of mare’s milk product, and reported outcomes related to gut microbiota and immune function. No meta-analysis was performed due to heterogeneity and absence of comparable intervention studies. Given the anticipated heterogeneity in study populations, supplementation protocols, microbiome assessment techniques, and reported outcomes, a structured narrative synthesis was expected to represent the principal method of evidence integration. The synthesis focused on identifying patterns of microbiome modulation, changes in beneficial or pathogenic microbial taxa, effects on inflammatory pathways and immune responses, and current knowledge gaps to guide future clinical research.

## 3. Results

### 3.1. Selected Studies

The systematic literature search identified a total of 116 records from four electronic databases: PubMed, Scopus, Web of Science and Embase. After exporting all references into a reference management system and removing duplicates, 64 studies underwent an initial title and abstract screening phase. During this stage, each record was independently assessed for relevance to the predefined eligibility criteria, focusing on (i) populations affected by COVID-19 or related inflammatory or post-infectious conditions, (ii) exposure to mare’s milk or mare’s milk-derived products, and (iii) reporting of outcomes related to gut microbiome composition, gastrointestinal function, or immune modulation. A further 42 studies were excluded due to irrelevance to mare’s milk interventions, absence of mare’s milk exposure, exclusive focus on unrelated nutritional or probiotic interventions without comparison to mare’s milk, and lack of extractable clinical or mechanistic data relevant to gut microbiome restoration or immune outcomes.

Following title and abstract screening, 22 articles were selected for full-text review, of which only 14 full-text manuscripts were accessible. Among the 14 available full-text articles, 6 were excluded due to failure to meet eligibility criteria, resulting in Eight studies meeting all predefined inclusion criteria and being included in the final synthesis. The complete selection process is illustrated in the PRISMA flow diagram (Figure S1).

### 3.2. Characteristics of Included Studies

A total of eight studies met the inclusion criteria and were included in the review [9,18,19,20,21,22,23,24]. Of these, five studies investigated gut microbiota alterations associated with COVID-19 infection or post-COVID conditions [9,18,19,20,21] (Table 1), while three studies examined the microbiological, immunomodulatory, and anti-inflammatory properties of mare’s milk or fermented mare’s milk (koumiss), providing evidence linking COVID-19-associated gut dysbiosis with the potential microbiome-restorative and immune-modulating effects of mare’s milk-based products [22,23,24] (Table 2).

For instance, Yeoh et al. evaluated 100 hospitalized adults with COVID-19 and demonstrated that disease severity was associated with depletion of beneficial commensal bacteria, including *Faecalibacterium prausnitzii*, *Eubacterium rectale*, and *Bifidobacterium* species, alongside enrichment of opportunistic pathogens [18]. Similarly, Zuo et al. conducted a longitudinal analysis of hospitalized COVID-19 patients and reported persistent alterations in gut microbial composition throughout hospitalization [19]. Liu et al. investigated patients with post-acute COVID-19 syndrome and found that gut dysbiosis persisted for up to six months after infection, with reduced microbial diversity and depletion of anti-inflammatory bacterial taxa associated with ongoing symptoms [9]. Two additional studies expanded these observations to pediatric and maternal–infant populations, including Romani et al.’s work, which reported distinct gut microbial signatures in children with SARS-CoV-2 infection compared with healthy controls, suggesting that COVID-19 may influence microbiome development early in life [20]. Likewise, Ignatyeva et al. demonstrated that maternal COVID-19 during pregnancy was associated with alterations in the infant gut microbiome during the first year of life, supporting the possibility of transgenerational effects of SARS-CoV-2-associated dysbiosis [21].

Also, three mechanistic studies investigated the biological properties of mare’s milk and koumiss, such as that by Abdel-Salam et al., which utilized a rat model of mercury-induced toxicity and found that supplementation with high-fiber probiotic fermented mare’s milk reduced oxidative stress, attenuated tissue injury, and improved biochemical markers of inflammation [22]. Tang et al. characterized the microbiota of traditional koumiss and identified diverse populations of lactic acid bacteria and yeasts, including *Lactobacillus* and *Lactococcus* species, highlighting the probiotic potential of fermented mare’s milk [23]. A more recent study by Wang et al. employed metagenomic approaches in an experimental model and demonstrated that mare’s milk supplementation increased microbial diversity, promoted beneficial bacterial taxa, and reduced pro-inflammatory cytokines, including interleukin-6 (IL-6), tumor necrosis factor-alpha (TNF-α), and interleukin-1β (IL-1β), while enhancing anti-inflammatory pathways [24].

The included studies consistently demonstrated that COVID-19 is associated with substantial disturbances in gut microbial composition that may persist beyond acute infection. Although no clinical studies directly evaluated mare’s milk supplementation in patients with COVID-19 or long COVID, mechanistic evidence suggests that mare’s milk and koumiss possess probiotic, anti-inflammatory, and microbiome-modulating properties that may be relevant for restoring gut microbial homeostasis and supporting immune recovery following COVID-19-associated dysbiosis.

### 3.3. Effects of COVID-19 on the Gut Microbiome in Children and Pregnant Women

Romani et al. evaluated gut microbiota profiles in children with SARS-CoV-2 infection and compared them with those of healthy controls. The study demonstrated that COVID-19 was associated with significant alterations in gut microbial composition, characterized by distinct microbial signatures and reduced microbiota stability [20]. Children with SARS-CoV-2 infection exhibited changes in bacterial diversity and taxonomic abundance, suggesting that even mild or asymptomatic infection may disrupt gut microbial homeostasis. Furthermore, microbiome alterations were associated with immune-related pathways, supporting the existence of bidirectional interactions between the gut microbiota and host immune responses during pediatric COVID-19. These findings indicate that SARS-CoV-2 infection can affect gut microbial development during childhood, a critical period for immune maturation and establishment of a stable intestinal microbiome.

The effects of COVID-19 on microbiome development were further explored by Ignatyeva et al., who investigated mother–infant pairs in which mothers had contracted COVID-19 during pregnancy [21]. Using 16S rRNA sequencing, the authors compared the gut microbiota of infants exposed to SARS-CoV-2 in utero with that of unexposed controls. Infants born to mothers with COVID-19 exhibited significantly reduced microbial diversity and richness, together with increased inter-individual variability, suggesting impaired establishment and reduced stability of the early-life gut microbiome. Notably, the observed alterations were independent of the trimester during which maternal infection occurred, indicating that prenatal exposure to SARS-CoV-2 may influence infant gut microbial development regardless of the timing of infection during pregnancy. The authors proposed that disruption of the maternal microbiome during COVID-19 may contribute to altered microbial transmission and colonization in offspring.

These studies suggest that the impact of COVID-19 on the gut microbiome extends beyond infected adults and may affect vulnerable populations, including children and infants exposed during gestation. The consistent findings of reduced microbial diversity, altered bacterial community structure, and potential disruption of normal microbiome maturation support concerns regarding the long-term consequences of SARS-CoV-2-associated dysbiosis during critical developmental periods. These observations further highlight the importance of investigating nutritional and microbiome-targeted interventions aimed at restoring gut microbial homeostasis in pediatric and perinatal populations affected by COVID-19.

### 3.4. Effects of Mare’s Milk on Gut Microbial Diversity and Composition

Some of the included studies investigated the mechanistic effects of mare’s milk on the microbiome suggesting that mare’s milk and its fermented derivative, koumiss, possess microbiome-modulating properties that may contribute to the restoration and maintenance of gut microbial homeostasis. Although direct clinical studies evaluating the effects of mare’s milk on the gut microbiota of patients with COVID-19 were not identified, available experimental and microbiological evidence indicates that mare’s milk contains bioactive components and microbial communities capable of influencing intestinal microbial diversity and composition.

For instance, Tang et al. characterized the microbial composition of traditional koumiss and demonstrated the presence of diverse populations of lactic acid bacteria and yeasts [23]. The dominant bacterial taxa included members of the genera *Lactobacillus* and *Lactococcus*, which are widely recognized for their probiotic properties and their role in maintaining intestinal health. In addition, the authors identified a complex microbial ecosystem that contributes to the fermentation process and production of organic acids, creating an environment that supports the growth of beneficial microorganisms while inhibiting potential pathogens. These findings suggest that koumiss may serve as a natural source of probiotic microorganisms capable of positively influencing gut microbial composition.

Further evidence was provided by Wang et al., who employed metagenomic analyses to investigate the effects of mare’s milk supplementation on the intestinal microbiota in an experimental model [24]. Mare’s milk administration was associated with increased microbial diversity and enrichment of bacterial taxa linked to anti-inflammatory and metabolic functions. The study reported shifts in microbial community structure favoring beneficial microorganisms while reducing the relative abundance of taxa associated with intestinal inflammation. Functional pathway analyses further suggested enhanced microbial activities related to immune regulation, nutrient metabolism, and maintenance of intestinal barrier integrity. These findings indicate that mare’s milk may promote a more balanced and resilient gut microbiome through both compositional and functional modifications.

Although Abdel-Salam et al. primarily investigated the protective effects of high-fiber probiotic fermented mare’s milk against mercury-induced toxicity in rats, the study also supports a role for fermented mare’s milk in maintaining intestinal microbial balance [22]. The probiotic formulation contributed to improvements in physiological and inflammatory parameters, suggesting that modulation of the gut ecosystem may have contributed to the observed protective effects. While direct microbiome sequencing was not performed, the findings are consistent with the established capacity of probiotic fermented dairy products to support beneficial microbial populations and suppress dysbiosis.

It can thus be concluded that mare’s milk and koumiss contain diverse probiotic microorganisms and bioactive compounds capable of influencing gut microbial ecology. Reported effects include increased microbial diversity, enrichment of beneficial bacterial taxa, promotion of metabolically favorable microbial functions, and suppression of potentially harmful microorganisms. These microbiome-modulating properties are particularly relevant in the context of COVID-19, where persistent reductions in microbial diversity and depletion of beneficial commensal bacteria have been consistently reported. Although direct evidence in COVID-19 populations remains unavailable, the observed effects of mare’s milk on microbial composition provide a plausible mechanistic basis for its potential use as a nutritional strategy to support gut microbiome restoration following SARS-CoV-2 infection.

### 3.5. Impact of Mare’s Milk on Immune Function and Inflammatory Markers

The included mechanistic studies suggest that mare’s milk and fermented mare’s milk products exert immunomodulatory and anti-inflammatory effects that may be relevant to conditions characterized by immune dysregulation and chronic inflammation, including post-COVID-19 syndrome. Although no clinical studies directly evaluated immune outcomes following mare’s milk supplementation in patients with COVID-19, experimental evidence indicates that mare’s milk can influence inflammatory pathways and host immune responses through both direct biological effects and microbiome-mediated mechanisms. The strongest evidence was provided by Wang et al., who investigated the effects of mare’s milk supplementation using metagenomic and immunological analyses [24]. Mare’s milk administration was associated with significant reductions in pro-inflammatory cytokines, including interleukin-6 (IL-6), tumor necrosis factor-alpha (TNF-α), and interleukin-1β (IL-1β), which are key mediators of systemic inflammation. In addition, the study demonstrated enhancement of anti-inflammatory immune pathways and favorable alterations in microbial metabolic functions associated with immune regulation. These findings suggest that mare’s milk may contribute to the attenuation of excessive inflammatory responses while promoting immune homeostasis through interactions between the gut microbiota and the host immune system.

Supporting evidence was reported by Abdel-Salam et al. in an experimental rat model of mercury-induced toxicity [22]. Animals receiving high-fiber probiotic fermented mare’s milk exhibited significant improvements in biochemical and histopathological markers of tissue injury compared with untreated controls. Supplementation was associated with reduced oxidative stress and attenuation of inflammatory damage in hepatic and renal tissues. Although specific cytokine profiles were not assessed, the observed reductions in tissue injury and improvements in antioxidant defense mechanisms support the anti-inflammatory potential of fermented mare’s milk. The authors proposed that the probiotic components of the fermented product contributed to the protective effects by mitigating inflammation and enhancing physiological resilience.

Tang et al. further provided indirect evidence supporting the immunomodulatory properties of mare’s milk-derived products through characterization of the microbial composition of traditional koumiss [23]. The study identified abundant populations of lactic acid bacteria and yeasts, including species belonging to the genera *Lactobacillus* and *Lactococcus*. These microorganisms are widely recognized for their capacity to modulate mucosal immunity, enhance intestinal barrier function, and influence cytokine production through interactions with the gut-associated lymphoid tissue. The presence of these probiotic microorganisms suggests a potential mechanism through which koumiss may contribute to immune regulation and maintenance of intestinal homeostasis; indicating that mare’s milk and fermented mare’s milk products may exert beneficial effects on immune function by reducing pro-inflammatory signaling, enhancing anti-inflammatory responses, and supporting microbiome-mediated immune regulation. These findings are particularly relevant in the context of COVID-19, where persistent immune activation and elevated inflammatory markers have been linked to disease severity and the development of post-acute sequelae. While the current evidence is limited to experimental and mechanistic studies, the observed effects on inflammatory pathways and immune homeostasis provide a biologically plausible rationale for investigating mare’s milk as a nutritional strategy to potentially support recovery from COVID-19-associated immune dysfunction and gut microbiome disturbances.

### 3.6. Effects on Gastrointestinal Health

Evidence regarding the effects of mare’s milk on gastrointestinal health and clinical outcomes was derived primarily from experimental studies, as no clinical trials evaluating mare’s milk supplementation in patients with COVID-19 or post-acute COVID-19 syndrome were identified. Nevertheless, the included studies suggest that mare’s milk and fermented mare’s milk products possess properties that may support intestinal health through modulation of the gut microbiota, reduction in inflammation, and enhancement of intestinal homeostasis. Abdel-Salam et al. investigated the effects of high-fiber probiotic fermented mare’s milk in a rat model of mercury-induced toxicity [22]. Animals receiving fermented mare’s milk demonstrated significant improvements in physiological and biochemical parameters compared with untreated controls. Histopathological analyses revealed reduced tissue damage in the liver and kidneys, accompanied by improvements in antioxidant defenses and attenuation of toxic effects. Although gastrointestinal outcomes were not the primary endpoint, the findings suggest that fermented mare’s milk may contribute to improved systemic health by mitigating inflammation and supporting gut-associated protective mechanisms.

Additional evidence was provided by Wang et al., who demonstrated that mare’s milk supplementation was associated with favorable alterations in gut microbial composition and function [24]. Metagenomic analyses indicated enrichment of beneficial microbial taxa and activation of metabolic pathways involved in intestinal homeostasis and immune regulation. These microbiome changes were accompanied by reductions in inflammatory markers, suggesting that mare’s milk may help maintain gastrointestinal integrity through modulation of the gut–immune axis. Improved microbial diversity and enhanced abundance of beneficial bacteria are generally considered indicators of a healthier intestinal environment and may contribute to improved gastrointestinal function.

Tang et al. characterized the microbial composition of traditional koumiss and identified abundant populations of probiotic lactic acid bacteria and yeasts capable of producing organic acids and other bioactive metabolites [23]. These microorganisms are known to support gastrointestinal health by promoting colonization resistance against pathogenic organisms, enhancing mucosal barrier function, and facilitating the production of short-chain fatty acids and other metabolites involved in intestinal homeostasis. The presence of these microbial communities provides a plausible biological basis for the traditionally reported digestive benefits of koumiss.

Although direct clinical evidence remains limited, the findings from the included studies suggest that mare’s milk and koumiss may support gastrointestinal health through multiple complementary mechanisms, including enhancement of microbial diversity, enrichment of beneficial bacterial populations, reduction in inflammation, and promotion of intestinal barrier integrity. These effects may be particularly relevant in the context of COVID-19, where gastrointestinal symptoms, persistent gut dysbiosis, and disruption of the gut–immune axis have been reported during both acute infection and the post-COVID recovery period.

Importantly, none of the included studies directly evaluated clinical outcomes such as gastrointestinal symptom resolution, quality of life, hospitalization rates, or recovery from long COVID following mare’s milk supplementation. Consequently, current evidence supporting clinical benefits remains indirect and mechanistic. While the observed effects on gut microbiota composition and inflammatory pathways provide a strong biological rationale for potential therapeutic investigation, well-designed human studies are needed to determine whether these microbiome-related changes translate into meaningful clinical improvements in patients recovering from COVID-19.

### 3.7. Safety, Tolerability, and Adverse Effects

None of the identified studies evaluated safety outcomes in patients with COVID-19 or post-acute COVID-19 syndrome, and adverse event reporting was generally sparse. However, in the experimental study by Abdel-Salam et al., administration of high-fiber probiotic fermented mare’s milk for six weeks was not associated with treatment-related toxicity or adverse physiological effects [22]. Instead, supplemented animals demonstrated improvements in biochemical and histopathological parameters compared with untreated controls. Similarly, Wang et al. reported no significant adverse effects associated with mare’s milk supplementation in their experimental model, with treated animals exhibiting favorable microbiome and inflammatory profiles throughout the study period [24]. Tang et al. focused on the microbiological characterization of traditional koumiss and did not assess clinical safety outcomes, but the beverage was found to contain diverse populations of lactic acid bacteria and yeasts commonly associated with fermented dairy products and probiotic activity [23]. The evidence suggests that mare’s milk and fermented mare’s milk products are generally well tolerated in experimental settings, with no significant adverse effects reported in the included studies. However, the absence of human clinical trials and limited safety reporting highlight the need for future studies incorporating systematic assessment of tolerability and adverse events, particularly in individuals recovering from COVID-19.

### 3.8. Assessment of Quality and Risk of Bias of Included Studies

The methodological quality of the included studies was generally moderate to high, with an overall low-to-moderate risk of bias. Among the five observational studies evaluating COVID-19-associated gut microbiota alterations, NOS assessments indicated that most studies were of high methodological quality. The prospective cohort studies by Yeoh et al. and Liu et al. exhibited the lowest risk of bias due to well-defined populations, comprehensive microbiome analyses, and adequate follow-up periods. Zuo et al.’s work was limited primarily by its small sample size, while the studies of Romani et al. and Ignatyeva et al. were susceptible to residual confounding arising from factors known to influence gut microbiota composition, including age, diet, antibiotic exposure, breastfeeding practices, and environmental influences (Table 3).

Similarly, the mechanistic studies evaluating mare’s milk and koumiss demonstrated generally acceptable methodological quality (Table 4). For example, Abdel-Salam et al. and Wang et al. employed controlled experimental designs and reported objective biological outcomes; however, incomplete reporting of randomization and blinding procedures resulted in a moderate risk of bias. Tang et al.’s work was a laboratory-based microbiological characterization study and therefore was not suitable for formal assessment using conventional clinical risk-of-bias tools. Nevertheless, the study employed appropriate sampling, sequencing, and analytical methodologies and was considered to have a low risk of methodological bias. Generally, the evidence base was limited by the absence of randomized clinical trials directly evaluating mare’s milk supplementation in patients with COVID-19 or post-COVID conditions. Consequently, while the included studies provide important mechanistic and observational evidence, the strength of causal inferences remains limited.

## 4. Discussion

This systematic review analyzed evidence from studies investigating COVID-19-associated gut microbiome alterations and the microbiome-modulating properties of mare’s milk and fermented mare’s milk products. Importantly, the review should be interpreted as hypothesis-generating rather than evidence-confirming, as the included studies did not directly evaluate the effects of mare’s milk or fermented mare’s milk in children or pregnant women recovering from COVID-19. Consequently, the proposed biological benefits discussed below are based on indirect and mechanistic evidence and should be interpreted as hypotheses requiring future clinical validation rather than established therapeutic effects. However, the included studies consistently demonstrate that SARS-CoV-2 infection is associated with significant disruption of gut microbial composition, characterized by reduced microbial diversity, depletion of beneficial commensal bacteria, and enrichment of opportunistic pathogens. These findings align with previous metagenomic and cohort studies reporting decreased abundance of SCFA-producing taxa such as *Faecalibacterium prausnitzii* and *Eubacterium rectale*, alongside enrichment of inflammatory or opportunistic organisms including *Enterococcus* and *Streptococcus* species in both acute and post-acute COVID-19 phases [25,26]. Importantly, these microbial alterations have been associated with systemic inflammation and disease severity, supporting the involvement of a gut-lung axis in COVID-19 pathophysiology [27]. Furthermore, longitudinal evidence indicates that microbial dysbiosis may persist for months following infection, particularly in individuals with post-acute sequelae of SARS-CoV-2 infection [9].

Other than adult populations, emerging evidence suggests including Romani et al., suggested that COVID-19-associated microbial disruption may extend to vulnerable developmental stages, including childhood during which SARS-CoV-2 infection is reported to be associated with altered microbial composition and reduced microbial stability during a critical period of immune maturation [20]. In parallel, Ignatyeva et al., reported an association between maternal infection during pregnancy and altered infant gut microbial colonization patterns, suggesting potential indirect or vertical transmission effects influencing early-life microbiome development [21]. Given the established role of early microbial colonization in immune programming and long-term metabolic health, such perturbations may have broader developmental implications [28,29].

Given the central role of the gut microbiome in immune and metabolic regulation, nutritional strategies aimed at restoring microbial homeostasis have gained increasing attention. Diet is a major determinant of microbial diversity and function, particularly through modulation of SCFA-producing bacteria, enhancement of epithelial barrier integrity, and regulation of inflammatory signaling pathways [2,16]. Functional foods containing prebiotics, probiotics, and bioactive compounds have therefore been proposed as potential adjunctive strategies for potential support of microbiome restoration following infection or inflammation [6,17].

Within this context, mare’s milk has attracted interest due to its unique biochemical and microbiological composition [30,31]. Compared with bovine milk, mare’s milk more closely resembles human milk in terms of lactose content, whey-to-casein ratio, and abundance of bioactive compounds, including lactoferrin, lysozyme, immunoglobulins, vitamins, and antioxidant molecules [7,30]. These components are implicated in antimicrobial defense, immune modulation, and intestinal barrier support. In addition, fermented mare’s milk products such as koumiss contain diverse populations of lactic acid bacteria and yeasts, including *Lactobacillus* and *Lactococcus* species, which may exert probiotic effects through competitive exclusion of pathogens and enhancement of mucosal immunity [32,33].

Mechanistically, mare’s milk and fermented mare’s milk products (e.g., koumiss) may influence gut microbial ecology through multiple complementary pathways. Its oligosaccharide fraction exhibits structural similarity to human milk oligosaccharides and may act as a selective substrate for beneficial taxa such as Bifidobacterium and *Lactobacillus*, thereby supporting SCFA production and intestinal barrier integrity [7]. This is particularly relevant in COVID-19, where depletion of SCFA-producing bacteria and persistent dysbiosis have been consistently reported [9,18].

In addition, bioactive proteins in mare’s milk, including lactoferrin, lysozyme, and immunoglobulins, exert antimicrobial and immunoregulatory effects through multiple mechanisms including iron sequestration, membrane disruption of pathogens, and modulation of cytokine signaling [34,35]. Fermentation further enhances bioactivity by generating peptides with antioxidant and immunomodulatory properties [36]. Collectively, these mechanisms suggest a biologically plausible capacity for koumiss to support microbiome resilience and immune homeostasis.

Experimental evidence further supports these biological properties, and showed that koumiss supplementation or fermented derivatives may improve microbial diversity, enhance beneficial bacterial taxa, and reduce inflammatory cytokine expression, including IL-6, TNF-α, and IL-1β [24]. Additional animal studies indicate reductions in oxidative stress and tissue injury following administration of fermented mare’s milk, suggesting systemic anti-inflammatory effects likely mediated through gut-immune interactions [22]. However, no human clinical trials have yet evaluated these effects in COVID-19 populations.

The relevance of these findings may be particularly pronounced in pediatric and pregnant populations, where microbiome stability is critical for immune development and maternal-fetal health. Early-life microbial colonization is highly sensitive to environmental perturbations, and disruptions during this period have been associated with long-term risks of immune-mediated and metabolic disease [37]. Similarly, maternal infection during pregnancy may alter infant microbial development through changes in maternal microbiota, immune signaling, and microbial transmission pathways [38,39]. Although koumiss has not been studied in these populations in the context of COVID-19, its composition suggests potential biological relevance, warranting careful investigation in future studies.

Clinically, persistent gut dysbiosis has been increasingly implicated in post-acute COVID-19 syndrome, suggesting that changed microbial composition may contribute to ongoing symptoms through reduced SCFA production, impaired gut barrier integrity, and chronic low-grade inflammation [16]. These mechanisms provide a plausible link between microbiome disruption and systemic manifestations of long COVID, including fatigue and neurocognitive dysfunction. Nutritional strategies aimed at restoring microbial balance, including probiotics and fermented foods, have therefore gained increasing scientific interest [6].

Several microbiome-targeted nutritional interventions, including probiotics, kefir, other fermented dairy products, and human milk oligosaccharides, have been investigated for their potential to restore gut microbial balance following infection or inflammation. Probiotics primarily provide beneficial microorganisms, whereas fermented dairy products such as kefir supply both live microbes and fermentation-derived bioactive metabolites. Human milk oligosaccharides function mainly as selective prebiotics that promote the growth of beneficial bacteria, particularly Bifidobacterium species. Compared with these interventions, mare’s milk possesses a distinctive combination of naturally occurring oligosaccharides, bioactive proteins (including lactoferrin, lysozyme, and immunoglobulins), and, in fermented preparations such as koumiss, diverse lactic acid bacteria. These compositional characteristics provide a biological rationale for further investigation; however, there is currently no direct clinical evidence demonstrating that mare’s milk is more effective than other microbiome-targeted nutritional interventions for restoring gut microbiome function following COVID-19. Within this framework, koumiss may represent a promising nutritional candidate due to its combined prebiotic, probiotic, and bioactive properties. However, the current evidence remains indirect and largely mechanistic, with no clinical trials evaluating its effects in COVID-19 or long COVID populations. Therefore, koumiss should currently be considered an exploratory intervention with biological plausibility rather than an established therapeutic option.

### 4.1. Future Perspectives

The findings of this review provide a biological rationale for future investigation rather than evidence supporting the clinical use of mare’s milk in individuals recovering from COVID-19. Well-designed randomized controlled trials are needed to determine whether the microbiome-modulating and immunoregulatory properties observed in experimental and indirect studies translate into clinically meaningful benefits. The trials should employ standardized mare’s milk and fermented mare’s milk formulations with clearly defined composition, processing methods, and dosing regimens to improve reproducibility and facilitate comparisons across studies. They should also include appropriate comparator groups, such as standard care or placebo/controlled interventions, and should incorporate predefined safety monitoring, particularly in children and pregnant women. Dose–response studies are also warranted to establish the optimal quantity, duration, and timing of supplementation.

Given the unique physiological characteristics of children and pregnant women, dedicated clinical studies in these populations are essential rather than extrapolating findings from adult cohorts. In addition to conventional clinical outcomes, future studies should incorporate longitudinal microbiome assessments, including measures of microbial diversity and community composition, together with immunological endpoints such as inflammatory and regulatory cytokines. Clinically relevant outcomes should include symptom severity, time to clinical recovery, gastrointestinal symptoms, duration of post-COVID-19 manifestations, and quality-of-life measures, where appropriate. Also, personalized nutrition approaches that account for baseline microbiome composition, dietary patterns, age, pregnancy status, and host metabolic characteristics may help identify individuals most likely to benefit from mare’s milk-based interventions. These studies will be necessary to determine whether the biological plausibility identified in the current review translates into safe and effective clinical applications.

### 4.2. Limitations

The study has several limitations that should be acknowledged. First, no clinical studies directly evaluated the effects of mare’s milk such as koumiss in children or pregnant women recovering from COVID-19. Consequently, this review is hypothesis-generating rather than evidence confirming, and any proposed benefits are based on indirect and mechanistic evidence rather than direct clinical observations. Second, the limited number of studies focused on mare’s milk, together with the reliance on experimental models and studies conducted in non-COVID populations, restricts the strength of causal inference and limits the applicability of the findings to post-COVID recovery. Third, substantial heterogeneity in study design, study populations, interventions, and outcome measures precluded a robust quantitative synthesis. Fourth, although five major international databases were searched, regional databases from countries where mare’s milk is traditionally consumed (e.g., Central Asia, Russia, Mongolia, and China) were not included. Consequently, relevant studies published in local journals or regional databases may have been missed, introducing potential publication and language bias. In addition, safety data in human populations remain insufficient, particularly in vulnerable groups such as pregnant women and children. Future research should prioritize well-designed randomized controlled trials evaluating mare’s milk and koumiss in post-COVID populations using standardized interventions and integrated multi-omics approaches. Particular attention should be given to pediatric and maternal cohorts, where persistent microbiome disruption may have long-term developmental implications. Such studies are needed to determine whether the biologically plausible mechanisms identified in experimental and indirect studies translate into clinically meaningful benefits, rather than assuming that these effects occur in humans recovering from COVID-19.

## 5. Conclusions

This systematic review highlights the growing evidence that COVID-19 is associated with persistent alterations in gut microbial composition, characterized by reduced microbial diversity, depletion of beneficial commensal microorganisms, and sustained immune dysregulation. These disturbances may be particularly important in vulnerable populations such as children and pregnant women, in whom the gut microbiome plays a critical role in immune maturation, metabolic regulation, and long-term health outcomes. Although no clinical studies directly evaluated the effects of mare’s milk or fermented mare’s milk products in individuals recovering from COVID-19, the available mechanistic and experimental evidence suggests that koumiss possesses several biological properties that may support potential microbiome restoration and immune recovery. Its high content of bioactive proteins, oligosaccharides, antioxidants, and probiotic microorganisms, particularly in fermented products such as koumiss, provides a plausible mechanistic basis for promoting beneficial microbial populations, improving microbial diversity, reducing inflammatory signaling, and supporting intestinal barrier integrity.

However, the current evidence remains indirect and is derived primarily from observational studies of COVID-19-associated dysbiosis and experimental investigations of mare’s milk in non-COVID settings. Consequently, koumiss cannot currently be recommended as an evidence-based therapeutic intervention for post-COVID gut dysbiosis or long COVID. Rather, it should be regarded as a promising nutritional strategy with substantial biological plausibility that warrants further investigation. Future research should prioritize well-designed randomized controlled trials evaluating koumiss and fermented mare’s milk products in pediatric, maternal, and post-COVID populations, incorporating comprehensive microbiome profiling, immunological assessments, and clinically relevant outcomes. Such studies will be essential to determine whether the favorable microbiome-modulating and immunoregulatory effects observed in experimental models translate into meaningful clinical benefits for individuals recovering from SARS-CoV-2 infection.

## Figures and Tables

**Table 1:** Characteristics of included studies investigating gut microbiota alterations associated with COVID-19.

Author (Year)	Country	Study Design	Study Population	Sample Size	Participant Characteristics	COVID-19 Status	Main Microbiome Findings	Follow-Up
Yeoh et al. (2021) [18]	China (Hong Kong)	Prospective cohort study	Hospitalized adult patients with confirmed COVID-19 and non-COVID controls	178 patients	Adults hospitalized with varying disease severity	Acute COVID-19	Reduced abundance of beneficial commensals (*Faecalibacterium prausnitzii*, *Eubacterium rectale*, *Bifidobacterium* spp.) and enrichment of opportunistic pathogens; dysbiosis correlated with inflammatory markers and disease severity	Up to 30 days after disease resolution
Zuo et al. (2020) [19]	China (Hong Kong)	Longitudinal cohort study	Hospitalized COVID-19 patients	15 patients	Adults admitted with laboratory-confirmed COVID-19	Acute COVID-19	Significant alterations in gut microbiota characterized by depletion of beneficial bacteria and enrichment of opportunistic pathogens; dysbiosis persisted during hospitalization	Duration of hospitalization
Liu et al. (2022) [9]	China (Hong Kong)	Prospective cohort study	Patients recovering from COVID-19 and a healthy control sample	174 patients	Adults evaluated after recovery from acute infection	Post-acute COVID-19 syndrome (Long COVID)	Persistent gut dysbiosis associated with long COVID symptoms; reduced microbial diversity and depletion of anti-inflammatory bacterial species observed months after infection	Six months post-infection
Romani et al. (2022) [20]	Italy	Observational pediatric cohort study	Children with SARS-CoV-2 infection and a control sample	130 children	Pediatric population with confirmed infection or exposure	Acute and convalescent COVID-19	Distinct microbial signatures associated with SARS-CoV-2 infection; altered bacterial composition compared with healthy controls	Cross-sectional assessment
Ignatyeva et al. (2025) [21]	Russia	Retrospective mother–infant cohort study	Infants born to mothers with COVID-19 during pregnancy vs. healthy infants	138 Participants	Neonates and infants with prenatal exposure to maternal COVID-19	Prenatal COVID-19 exposure	Maternal COVID-19 was associated with altered infant gut microbiome composition and reduced abundance of beneficial microbial taxa during early life	First year of life

**Table 2:** Characteristics of included mechanistic studies evaluating mare’s milk.

Author (Year)	Country	Study Design	Population/Sample	Sample Size	Intervention/Exposure	Duration	Main Outcomes	Key Findings
Abdel-Salam et al. (2010) [22]	Saudi Arabia	Experimental animal study	Mercury-exposed rats	30 rats	High-fiber probiotic fermented mare’s milk	6 weeks	Oxidative stress, liver and kidney toxicity, histopathological changes	Fermented mare’s milk significantly reduced mercury-induced toxicity, improved antioxidant status, and attenuated tissue damage, suggesting anti-inflammatory and protective biological effects.
Tang et al. (2020) [23]	China	Microbiological and metagenomic analysis	Traditional koumiss samples collected from Inner Mongolia	14 koumiss samples	Characterization of koumiss microbiota and organic acid composition	Cross-sectional analysis	Microbial diversity, bacterial and yeast composition, organic acid profiles	Koumiss contained diverse lactic acid bacteria and yeasts, including *Lactobacillus*, *Lactococcus*, and *Kazachstania* species, supporting its potential probiotic properties and contribution to gut microbial modulation.
Wang et al. (2025) [24]	China	Experimental metagenomic study	Mouse model receiving mare milk supplementation	32 mice	Mare milk supplementation	4 weeks	Gut microbiota composition, inflammatory cytokines, microbial metabolic pathways	Mare milk increased microbial diversity, promoted beneficial bacterial taxa, reduced pro-inflammatory cytokines (including IL-6, TNF-α, and IL-1β), and enhanced anti-inflammatory pathways, suggesting a role in immune regulation through microbiome modulation.

**Table 3:** Quality assessment and risk of bias. Observational—Newcastle–Ottawa Scale.

Study	Selection (4)	Comparability (2)	Outcome (3)	Total Score	Quality Rating
Yeoh et al., 2021 [18]	4	2	3	9/9	High quality
Zuo et al., 2020 [19]	4	1	3	8/9	High quality
Liu et al., 2022 [9]	4	2	3	9/9	High quality
Romani et al., 2022 [20]	3	1	3	7/9	Moderate-to-high quality
Ignatyeva et al., 2025 [21]	4	1	3	8/9	High quality

**Table 4:** Quality assessment and risk of bias. A summary of SYRCLE assessments of experimental and mechanistic studies.

Study	Study Design	Main Strengths	Main Limitations	Overall Risk of Bias
Abdel-Salam et al. (2010) [22]	Experimental animal study	Controlled experimental design; objective biochemical and histopathological outcomes	Insufficient reporting of randomization and blinding procedures	Moderate
Tang et al. (2020) [23]	Microbiological characterization study	Robust microbial sequencing and analytical methods	No intervention group or clinical outcomes; limited translational applicability	N/A *
Wang et al. (2025) [24]	Experimental metagenomic study	Comprehensive microbiome and inflammatory analyses; objective outcome measures	Incomplete reporting of allocation concealment and blinding	Low to Moderate

* The SYRCLE risk-of-bias tool was applied only to experimental animal studies. Descriptive microbiological characterization studies (e.g., Tang et al. [23]) were considered not applicable for SYRCLE assessment.

## Data Availability

No new data were created or analyzed in this study. Data sharing is not applicable to this article.

## Supplementary Materials

The following supporting information can be downloaded at: https://www.mdpi.com/article/10.3390/biom16081203/s1, Figure S1: PRISMA flow diagram of study selection; Table S1: Detailed Search Strategies for PubMed, Scopus, and Web of Science.
