# Supplementary material for: Mare’s Milk for Gut Microbiome Restoration and Immune Recovery After COVID-19 in Children and Pregnant Women: A Hypothesis-Generating Systematic Review
Source: Biomolecules. 2026 Aug 17;16(8):1203. doi: 10.3390/biom16081203 (PMC13511651; doi:10.3390/biom16081203)
Supplement: Supplementary file 1 [file biomolecules-16-01203-s001.zip › biomolecules-4444461-supplementary.pdf]

biomolecules

MDPI

Table S1. Detailed Search Strategies for PubMed, Scopus, and Web of Science.

Database	Search Strategy
PubMed	(("mare's milk" OR "equine milk" OR "fermented mare's milk" OR kumis) AND ("gut microbiome" OR "gut microbiota" OR "intestinal microbiota" OR "microbial diversity" OR dysbiosis) AND ("immune response" OR inflammation OR immunomodulation) AND ("COVID-19" OR "SARS-CoV-2") AND (children OR pediatric OR pregnancy OR "maternal health"))
Scopus	TITLE-ABS-KEY(("mare's milk" OR "equine milk" OR "fermented mare's milk" OR kumis) AND ("gut microbiome" OR "gut microbiota" OR "intestinal microbiota" OR "microbial diversity" OR dysbiosis) AND ("immune response" OR inflammation OR immunomodulation) AND ("COVID-19" OR "SARS-CoV-2") AND (children OR pediatric OR pregnancy OR "maternal health"))
Web of Science	TS=(("mare's milk" OR "equine milk" OR "fermented mare's milk" OR kumis) AND ("gut microbiome" OR "gut microbiota" OR "intestinal microbiota" OR "microbial diversity" OR dysbiosis) AND ("immune response" OR inflammation OR immunomodulation) AND ("COVID-19" OR "SARS-CoV-2") AND (children OR pediatric OR pregnancy OR "maternal health"))

 https://doi.org/10.3390/biom16081203

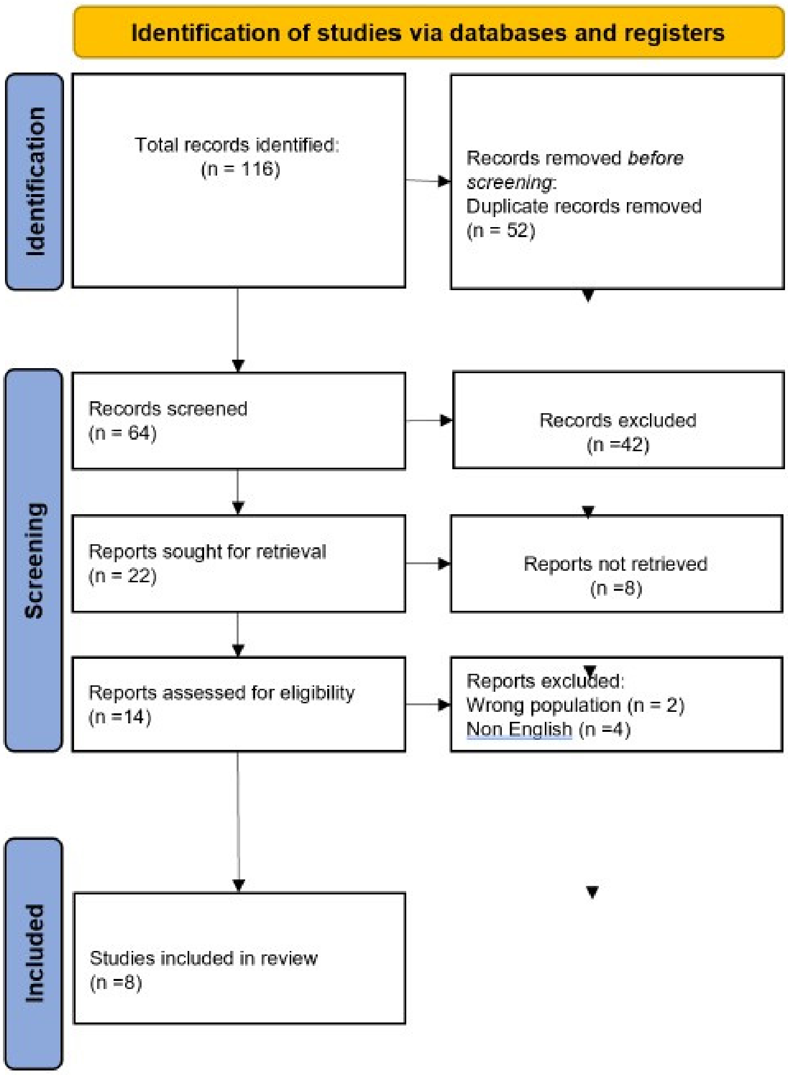

Figure S1. PRISMA flow diagram of study selection.
